# Preoperative, biopsy‐based assessment of the tumour microenvironment in patients with primary operable colorectal cancer

**DOI:** 10.1002/cjp2.143

**Published:** 2019-10-14

**Authors:** James H Park, Hester van Wyk, Donald C McMillan, Joanne Edwards, Clare Orange, Paul G Horgan, Campbell SD Roxburgh

**Affiliations:** ^1^ Academic Unit of Surgery, School of Medicine Dentistry and Nursing College of Medicine Veterinary and Life Sciences, University of Glasgow, Glasgow Royal Infirmary Glasgow UK; ^2^ Department of Experimental Therapeutics Institute of Cancer Sciences, College of Medicine Veterinary and Life Sciences, University of Glasgow Glasgow UK; ^3^ NHS Greater Glasgow & Clyde Biorepository Queen Elizabeth University Hospital Glasgow UK

**Keywords:** colorectal cancer, tumour microenvironment, stroma, biopsy, preoperative assessment, prognosis

## Abstract

The tumour microenvironment (TME) is recognised as an important prognostic characteristic and therapeutic target in patients with colorectal cancer (CRC). However, assessment generally utilises surgically resected specimens, precluding neoadjuvant targeting. The present study investigated the feasibility of intra‐epithelial CD3^+^ T‐lymphocyte density and tumour stroma percentage (TSP) assessment using preoperative colonoscopic biopsies from 115 patients who had undergone resection of stages I–III CRC, examining the relationship between biopsy and surgically resected specimen‐based assessment, and the relationship with cancer‐specific survival (CSS). High biopsy CD3^+^ density was associated with high CD3^+^ density in the invasive margin, cancer stroma and intra‐epithelial compartments of surgically resected specimens (area under the curve > 0.62, *p* < 0.05 for all) and with high Immunoscore. High biopsy TSP predicted high TSP in resected specimens (*p* = 0.001). Intra‐class correlation coefficient for both measures was >0.7 (*p* < 0.001), indicating excellent concordance between individuals. Biopsy CD3^+^ density (hazard ratio [HR] 0.23, *p* = 0.002) and TSP (HR 2.23, *p* = 0.029) were independently associated with CSS; this was comparable to the prognostic value of full section assessment (HR 0.21, *p* = 0.004, and HR 2.25, *p* = 0.033 respectively). These results suggest that assessment of the TME is comparable in biopsy and surgically resected specimens from patients with CRC, and biopsy‐based assessment could allow for stratification prior to surgery or commencement of therapy targeting the TME.

## Introduction

The prognosis of colorectal cancer and the need for adjuvant therapy are determined by pathological staging according to IUCC/AJCC TNM staging criteria. However, such staging is suboptimal, particularly given that increasing disease stage may not necessarily reflect increasing risk of cancer‐associated mortality 1. As such, there has been a concerted effort over the past decades to identify both molecular and pathological characteristics that may determine both prognosis and need for additional treatment.

Tumour microenvironment characteristics are important determinants of disease progression and survival. A pronounced local inflammatory cell infiltrate is synonymous with good prognosis across a number of solid organ cancers, with numerous scores based upon this premise described in patients with colorectal cancer 2, 3, 4. Of these, the Immunoscore©, a measure of CD3^+^ and CD8^+^ T‐lymphocyte density, has recently been internationally validated as a stage‐independent predictor of survival in stages I–III disease 4. Similarly, other components of the tumour microenvironment, such as the tumour‐associated stroma 5, hold additional and complementary prognostic value when examined in combination with the inflammatory cell infiltrate 6, 7.

In addition to determining prognosis, the tumour microenvironment may also aid in determining non‐surgical treatment of patients with colorectal cancer. Both the local immune response and the tumour‐associated stroma moderate response to chemotherapy and radiotherapy 8, 9, 10, 11, 12, 13. Furthermore, the tumour microenvironment may be a potential target for novel therapies, such as immune checkpoint blockade and TGF‐β inhibition, in its own right 14, 15, 16.

However, one limitation of the use of the tumour microenvironment to guide treatment is that methods described to date have generally relied on comprehensive assessment of the surgically resected specimen. Although aiding in the determination of prognosis, this limits the potential for selection of preoperative neoadjuvant therapies based upon tumour microenvironment characteristics. Furthermore, it also precludes the use of such measures to inform prognosis of patients with metastatic or locally advanced disease not amenable to surgical resection, as well as those with complete pathological response following neoadjuvant treatment.

One potential strategy to assess the tumour microenvironment prior to surgery is the use of preoperative endoscopic biopsies. Several groups have characterised the local inflammatory cell infiltrate in this manner, with a view to determining treatment‐related changes in immune cell infiltrates and predicting response to chemoradiotherapy in patients with rectal cancer 8, 9, 10, 11, 12, 17, 18. However, to date only three small studies (examining 31, 54 and 50 patients, respectively) have compared the local inflammatory cell infiltrate in biopsies and fully resected surgical specimens of patients without intervening neoadjuvant chemoradiotherapy 9, 10, 19. Furthermore, although previously examined in patients with oesophageal cancer 20, the feasibility of biopsy‐based assessment of the tumour stroma in patients with colorectal cancer remains to be fully determined. Therefore, whether biopsy‐based assessment of tumour microenvironment characteristics is directly comparable to surgically resected specimens, and whether such measures may also determine prognosis, remains to be determined.

The aim of the present study was to examine the feasibility of assessment of the tumour inflammatory cell infiltrate and stroma using endoscopy biopsy specimens in a cohort of patients undergoing potentially curative resection of stages I–III colorectal cancer without prior neoadjuvant therapy.

## Materials and methods

### Patients

Patients who had undergone resection of stages I–III colorectal cancer in Glasgow Royal Infirmary between 1997 and 2005, and in whom the tumour microenvironment had been characterised as part of a previous study 21, were included. Only patients who had undergone colonoscopy and biopsy with pathological confirmation of invasive colorectal adenocarcinoma within the biopsy specimen were included. Patients without biopsy evidence of invasion, who had received neoadjuvant chemoradiotherapy prior to surgery, or who underwent localised excision only were excluded.

Tumours were staged using the fifth edition of the tumour, node and metastases (TNM) classification, consistent with practice current in the United Kingdom during the study period 22. Patients were followed‐up for 5 years following surgery. Date and cause of death was crosschecked with the cancer registration system and the Registrar General (Scotland), with death records complete until 15 March 2013 that served as censor date. Cancer‐specific survival was measured from surgery until date of death from metastatic or recurrent colorectal cancer. The local institutional ethics committee approved the study.

### Assessment of tumour microenvironment

#### Surgically resected specimens

Using full sections from the surgically resected specimen, the density of mature (CD3^+^) and cytotoxic (CD8^+^) T‐lymphocytes within the invasive margin, cancer epithelium and stroma was semi‐quantitatively assessed at ×100 total magnification as absent or low (low), or moderate or high (high) as previously described 21. Assessment was based on a generalised overview of the full stained section rather than individual regions. The invasive margin was defined as ‘the interface between the host stroma and the invading edge are of a tumour’, excluding intra‐epithelial lymphocytes at the invasive edge, consistent with prior work by Klintrup *et al* 3. A composite immune‐infiltrate score, composed of density of CD3^+^ and CD8^+^ T‐lymphocytes within the invasive margin and intra‐epithelial compartments, was calculated ranging from Im0 (both low in both regions) to Im4 (both high in both regions).

The tumour‐associated stroma was assessed using tumour stroma percentage (TSP) as previously described 13. In brief, using H&E‐stained sections of the deepest point of invasion, the area of intra‐tumoural stroma was graded as low (≤50%) or high (>50%).

#### Biopsy specimens

Formalin‐fixed paraffin‐embedded blocks and H&E‐stained sections of endoscopic biopsy specimens taken at time of diagnosis were used. Sections 2.5 μm thick were cut and mounted on silanised slides before being dewaxed and rehydrated through graded alcohol. An autostainer (ThermoFisher Autostainer 480s) was used to perform staining. Antigen retrieval was carried out in a PT module (Thermo Fisher, Waltham, MA, USA) using ThermoFisher dewax/retrieve solution pH 9. Primary antibody (CD3^+^; 9107S, ThermoFisher) was applied for 20 min (1:100 dilution) at room temperature following antigen retrieval. Signal was amplified and visualised using the ThermoFisher Quanto kit and the diaminobenzidine (DAB) colour developer. Sections were converted to digital format using a Hamamatsu Nanozoomer (Welwyn Garden City, Hertfordshire, UK) at ×20 objective magnification, and visualisation performed using Slidepath Digital Image Hub, version 4.0.1 (Slidepath, Leica Biosystems, Milton Keynes, UK).

At ×200 total magnification, a maximum of three 0.6 mm × 0.6 mm (total area 0.36 mm^2^) high‐power fields (HPF) most representative of the whole section were identified, and the number of intra‐epithelial CD3^+^ T‐lymphocytes within each HPF counted and an average calculated. Areas of dysplastic or normal mucosa were excluded, as were T‐lymphocytes outside the epithelial compartment. This method was chosen as it was assumed to be comparable to the use of a tissue microarray, where assessment of three cores (0.6 mm diameter) has been considered comparable to full section analysis 23.

**Figure 1:**
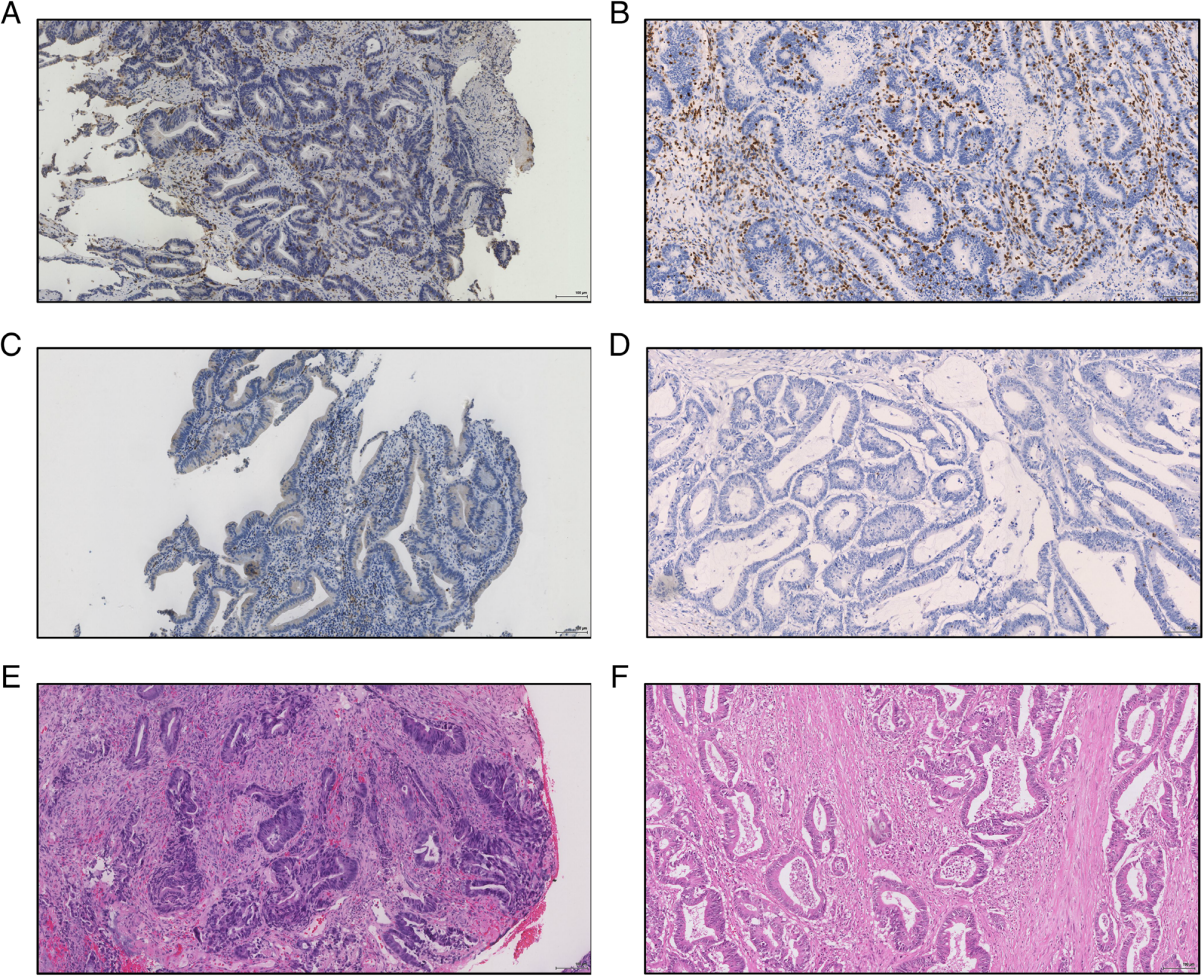
Examples of matched biopsy‐based and full section‐based assessments of the tumour microenvironment in patients with colorectal cancer (×200 total magnification). Panels A and B respectively display a biopsy specimen with high intra‐epithelial CD3^+^ density (122 cells/high powered field) and corresponding full section with high intra‐epithelial and stromal CD3^+^ density. Both specimens also display a low tumour stroma percentage. Panels C and D respectively display a biopsy specimen with low intra‐epithelial CD3^+^ density (9 cells/high powered field) and corresponding full section with low intra‐epithelial and stromal CD3^+^ density. Panels E and F display matched biopsy and full haematoxylin and eosin‐stained sections with high tumour stroma percentage.

Assessment of TSP using H&E‐stained biopsy sections was performed similarly to Courrech Staal *et al* 20. At ×100 total magnification, the proportion of intra‐tumoural stroma was graded as low (≤50%) or high (>50%). Assessment was performed in regions where tumour cells were present circumferentially, with mucinous deposits, necrosis and dysplastic or normal mucosa, or stromal fragments without any cancer cells, excluded. Both biopsy T‐lymphocyte and TSP were scored by a single examiner blinded to clinicopathological data (JHP), with independent co‐scoring of each by a blinded examiner (HvW and CSDR, respectively). Inter‐observer variability was examined using intra‐class correlation coefficient. Examples of biopsy CD3^+^ T‐lymphocyte staining and TSP assessment are displayed in Figure 1.

### Statistical analysis

The relationship between biopsy intra‐epithelial CD3^+^ T‐lymphocyte count and T‐lymphocyte density within the invasive margin, cancer stroma and intra‐epithelial compartment of full, surgically resected specimens was examined using Mann–Whitney *U*‐test and area under the curve (AUC) to identify optimal threshold for low/high density within biopsy specimens. Chi‐square analysis for linear trend was subsequently used to compare tumour microenvironment characteristics in biopsy and surgically resected specimens.

To further examine clinical utility of biopsy‐based assessment of the tumour microenvironment, the relationship with cancer‐specific survival was examined. Univariate Cox regression analysis was subsequently performed to calculate hazard ratios (HRs) and 95% CI. To assess whether biopsy assessment had comparable prognostic value to conventional, full‐section assessment, two multivariate models were created alongside other clinicopathological characteristics using a backwards stepwise method; the first model included biopsy intra‐epithelial CD3^+^ density and TSP, whereas the second model included assessment of CD3^+^ density and TSP using surgically resected specimens. Kaplan–Meier curves and log‐rank analysis were used to compare difference between groups. A *P* value ≤0.05 was considered statistically significant. All analyses were performed using SPSS version 22.0 for Mac (Armonk, NY, USA).

## Results

Matched biopsy and surgically resected specimens of 120 patients who underwent potentially curative resection of stages I–III colorectal cancer were retrieved. Five patients did not have adequate biopsy tissue for CD3^+^ staining, resulting in 115 patients being included in the study; clinicopathological characteristics are displayed in Table 1. About 91 patients (79%) had sufficient biopsy material to examine three HPFs; of the remaining patients, 12 had sufficient material for examination of two HPFs, and 12 for examination of one field. Mismatch repair status was available for 91 patients; 9 patients (10%) were MMR deficient. The intra‐class correlation coefficient for assessment of biopsy intra‐epithelial CD3^+^ density and TSP were 0.866 and 0.743, respectively (both *p* < 0.001), indicating excellent concordance.

**Table 1:** Clinicopathological characteristics of patients with primary operable colorectal cancer (*n* = 115)

Clinicopathological characteristics (*n* when data missing)	Patients (%)
Age	
<65	43 (37)
65–74	36 (31)
>74	36 (31)
Sex	
Male	61 (53)
Female	54 (47)
Adjuvant therapy	
No	80 (70)
Yes	35 (30)
Tumour site	
Colon	78 (68)
Rectum	37 (32)
TNM stage	
I	6 (5)
II	52 (45)
III	57 (50)
T stage	
1–2	12 (10)
3	73 (64)
4	30 (26)
N stage	
0	58 (50)
1	45 (39)
2	12 (11)
Differentiation	
Mod‐well	110 (96)
Poor	5 (4)
Venous invasion	
Absent	78 (68)
Present	37 (32)
Margin involvement	
Absent	110 (96)
Present	5 (4)
Peritoneal involvement	
Absent	85 (74)
Present	30 (26)
Tumour perforation	
Absent	113 (98)
Present	2 (2)
Mismatch repair status (*n* = 91)	
Competent	82 (90)
Deficient	9 (10)
CD3^+^ margin density (*n* = 114)	
Low	61 (53)
High	53 (47)
CD3^+^ stroma density	
Low	52 (45)
High	63 (55)
CD3^+^ cancer cell nest	
Low	77 (67)
High	38 (33)
CD8 margin density (*n* = 107)	
Low	59 (55)
High	48 (45)
CD8 stroma density (*n* = 110)	
Low	76 (69)
High	34 (31)
CD8 cancer cell nest (*n* = 110)	
Low	75 (65)
High	35 (30)
Immune cell density (*n* = 107)	
0	37 (35)
1–2	41 (38)
3	17 (16)
4	12 (11)
Tumour stroma percentage	
Low	90 (78)
High	25 (22)

The median biopsy intra‐epithelial CD3^+^ T‐lymphocyte count was 24 cells/HPF (interquartile range [IQR] 16–36, range 4–183). Tumours with a high CD3^+^ density within the invasive margin, stroma and intra‐epithelial compartments of the surgically resected specimen had a higher biopsy T‐lymphocyte count (all *p* < 0.05, Table 2). Receiving operating characteristic and AUC was used to determine threshold for biopsy CD3^+^ T‐lymphocyte count (Table 2, and see supplementary material, Figure S1); the optimal cut‐off for predicting high density within each region was identical and corresponded with the median (low <25 cells/HPF; high ≥25 cells/HPF). Biopsy density was most predictive of intra‐epithelial CD3^+^ density, with a sensitivity and specificity of 79 and 70%, and positive and negative predictive value of 57 and 87% (see supplementary material, Table S1). In addition to being associated with invasive margin, stromal and intra‐epithelial CD3^+^ density, high biopsy intra‐epithelial CD3^+^ density was also associated with intra‐epithelial CD8^+^ density and the combined CD3^+^/CD8^+^ assessment (both *p* < 0.001), and showed a trend towards an association with invasive margin and stromal CD8^+^ density (*p* = 0.07 and *p* = 0.058 respectively; Table 3).

**Table 2:** Relationship between biopsy intra‐epithelial CD3^+^ T‐cell count and full section assessment of CD3^+^ T‐cell density

		Biopsy CD3^+^ T‐cell count		
Full section CD3^+^ T‐cell density	*n*	Median CD3^+^ cell count (IQR, max)	*P* value*	AUC (95% CI)
	115	24 (16–36; 4–183)	–	–
CD3^+^ margin			0.024	0.622 (0.519–0.726)
Low	61	22 (13–32)		
High	53	27 (19–43)		
CD3^+^ stroma			0.005	0.651 (0.549–0.753)
Low	52	20 (14–29)		
High	63	27 (19–37)		
CD3^+^ intra‐epithelial			<0.001	0.773 (0.674–0.872)
Low	77	20 (13–27)		
High	38	34 (25–63)		

* Mann–Whitney *U*‐test.

**Table 3:** Relationship between biopsy and full section assessment of the tumour microenvironment

	Biopsy intra‐epithelial CD3^+^ T‐cell density		
Full section tumour microenvironment	Low (*n* = 61)	High (*n* = 53)	*P* value
CD3^+^ margin			0.045
Low	38	23	
High	23	30	
CD3^+^ stroma			0.003
Low	36	16	
High	26	37	
CD3^+^ intra‐epithelial			<0.001
Low	54	23	
High	8	30	
CD8^+^ margin			0.070
Low	35	24	
High	20	28	
CD8^+^ stroma			0.058
Low	44	32	
High	12	21	
CD8^+^ intra‐epithelial			<0.001
Low	48	27	
High	9	26	
Immunoscore			<0.001
0	27	10	
1–2	21	20	
3	5	12	
4	2	10	

	Biopsy stroma percentage		
	Low (*n =* 73)	High (*n* = 42)	*P* value
TSP			0.001
Low	64	26	
High	9	16	

Threshold: low <25 CD3^+^ T‐lymphocytes/HPF, high ≥25 CD3^+^ T‐lymphocytes/HPF.

Biopsy assessment of TSP was examined (Table 3) and found to be associated with full section assessment (*p* = 0.001). Although the negative predictive value of biopsy‐based assessment was high, the positive predictive value was low (90 and 38% respectively; see supplementary material, Table S1).

About 4 patients (44%) with MMR deficient cancer each had a high biopsy intra‐epithelial CD3^+^ density and biopsy TSP compared to 35 (43%) and 28 (34%) of patients with MMR competent colorectal cancer respectively. The small number of patients with MMR deficient colorectal cancer precluded meaningful statistical analysis of the relationship between MMR status and tumour microenvironment characteristics.

Median follow‐up of survivors was 136 months (range 89–193) with 33 cancer and 32 non‐cancer deaths. On univariate survival analysis, a high biopsy intra‐epithelial CD3^+^ density was associated with improved survival (HR 0.21, 95% CI 0.09–0.52, *p* = 0.001) whereas a high biopsy TSP was associated with reduced survival (HR 2.78, 95% CI 1.39–5.54, *p* = 0.004). The effect on survival was comparable to assessment of CD3^+^ density and TSP using surgically resected specimens (HR 0.22, 95% CI 0.08–0.64, *p* = 0.005, and HR 2.41, 95% CI 1.17–4.98, *p* = 0.018). On multivariate analysis (Table 4), biopsy CD3^+^ density (HR 0.23, *p =* 0.002) and biopsy TSP (HR 2.23, *p* = 0.029) were associated with survival independent of TNM stage, venous invasion and margin involvement. This was again comparable to the prognostic value of assessment using surgically resected specimens (see supplementary material, Table S2).

**Table 4:** Relationship between clinicopathological characteristics, biopsy assessment of the tumour microenvironment and cancer‐specific survival

Biopsy assessment	Cancer‐specific survival			
Clinicopathological characteristics	Univariate analysis	*P* value	Multivariate analysis	*P* value
Age (<65/65–74/>75)	1.17 (0.77–1.77)	0.471	–	–
Sex (female/male)	1.41 (0.70–2.84)	0.331	–	–
Adjuvant therapy (no/yes)	1.21 (0.59–2.51)	0.600	–	–
Tumour site (colon/rectum)	1.74 (0.86–3.50)	0.123	–	–
TNM stage (I/II/III)	2.49 (1.25–4.93)	0.009	2.24 (1.09–4.59)	0.029
Tumour differentiation (mod‐well/poor)	1.50 (0.40–6.29)	0.577	–	–
Venous invasion (no/yes)	3.34 (1.66–6.70)	0.001	2.22 (1.07–4.61)	0.033
Margin involvement (no/yes)	5.93 (2.05–17.11)	0.001	8.18 (2.52–26.55)	<0.001
Peritoneal involvement (no/yes)	1.69 (0.83–3.44)	0.147	–	–
Tumour perforation (no/yes)	3.10 (0.42–22.81)	0.266	–	–
Mismatch repair status (competent/deficient)	0.34 (0.05–2.49)	0.287	–	–
Biopsy T‐lymphocyte density (low/high)	0.21 (0.09–0.52)	0.001	0.23 (0.09–0.57)	0.002
Biopsy tumour stroma percentage (low/high)	2.78 (1.39–5.54)	0.004	2.23 (1.09–4.58)	0.029

**Figure 2:**
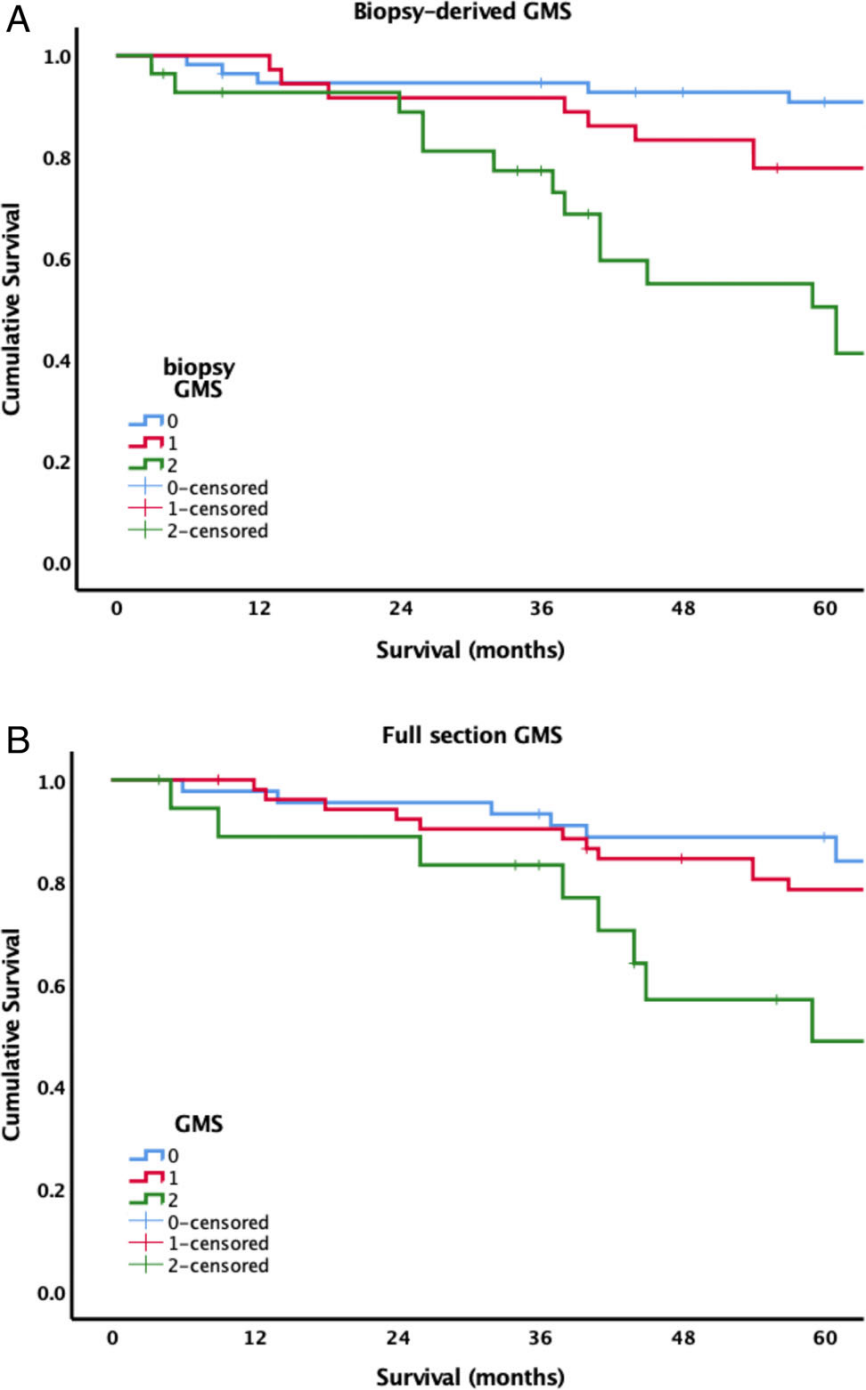
Comparison of (A) biopsy, and (B) full‐section based assessment of Glasgow Microenvironment Score in patients with primary operable colorectal cancer.

The Glasgow Microenvironment Score (GMS), composed of assessment of the local inflammatory cell infiltrate and TSP, has been shown to have greater prognostic value than either measure alone in patients with colorectal cancer 21, 22. As such, the feasibility of a biopsy‐based GMS (bGMS) was examined, and devised as follows: bGMS0 – high biopsy intra‐epithelial CD3^+^ density; bGMS1 – low biopsy intra‐epithelial CD3^+^ density and low biopsy TSP; bGMS2 – low biopsy intra‐epithelial CD3^+^ density and high biopsy TSP. This score was similar to full section assessment of the GMS in this cohort (Figure 2), and stratified survival of patients into three prognostic groups with five‐year survival of 92% (*n* = 53), 76% (*n* = 34) and 51% (*n =* 28), respectively (*p* < 0.001).

The biopsy specimens of patients in which there was a discrepancy between biopsy and full section intra‐epithelial CD3^+^ density were further examined (see supplementary material, Table S3). Seven patients were incorrectly classified as having low CD3^+^ density on the basis of biopsy assessment; of these, three biopsy specimens were fragmented and had small surface area, and two were of small foci of invasive cancer within adenoma or normal mucosa. The remaining two specimens showed no evident issues, suggesting that technical factors accounted for incorrect classification in five of seven cases. A total of 23 patients were incorrectly classified as high CD3^+^ density on the basis of biopsies. Of those with a biopsy CD3^+^ > 48 cells/HPF, only two were incorrectly classified as high density, with both biopsy specimens fragmented and with small surface area. Of the remaining 21 incorrectly classified patients, four biopsy specimens were fragmented/had small surface area, three had small foci of cancer within adenoma and 14 showed no evident technical issues.

## Discussion

The present study confirms that preoperative, endoscopic biopsy‐based assessment of the tumour microenvironment is representative of assessment within the surgically resected specimen and has comparable prognostic value. In addition to aiding in the preoperative staging of patients with colorectal cancer, biopsy‐based assessment may also identify patients who could potentially benefit from neoadjuvant strategies targeting the tumour microenvironment.

Recent interest in the molecular taxonomy of colorectal cancer has directed efforts to identify molecular subtype prior to surgery using biopsy‐derived tissue 24. Of interest, although epithelial cell‐derived subtypes appear to be robustly predicted using biopsy specimens, those which incorporate mesenchymal and immune cell derived components appear to be identified less reliably 24. Therefore, it is reassuring in the present study that biopsy intra‐epithelial CD3^+^ density correlated with full section assessment of the local inflammatory cell infiltrate across a number of different tumour regions. This is consistent with prior work proposing correlation of immune cell densities across different tumour subsites 21. However, although biopsy‐assessment of TSP correlated significantly with full section assessment, this association was not as robust. Indeed, this would suggest that, despite lumen‐derived biopsies being a reliable surrogate for assessment of the local inflammatory cell infiltrate, they may not as accurately predict TSP.

However, the present study relied on the use of retrospective, clinical‐grade biopsy specimens. Biopsy specimens vary not only in size and tissue quality, but also abundance of tumour tissue present 25. Whereas the only criterion for biopsies to be included in the present analysis was the presence of invasive malignancy, more rigorous criteria may result in more accurate assessment. For example, Saridaki *et al* reported that stringent selection criteria for biopsy sections (at least 20% of invasive malignancy present in the biopsy and at least six fragments present) increased concordance with full section analysis for mutational analysis 26. In the present study, it was apparent that technical factors related to biopsy specimen quality resulted in incorrect classification of patients, particularly those incorrectly classified as having low CD3^+^ T‐cell density. Furthermore, biopsy specimens of sufficient size to allow for at least three HPFs to be examined increased sensitivity and specificity (data not shown). Therefore, future studies should ensure that sufficient representative biopsy specimens are retrieved using a standardised approach, incorporating adequate targeting of the tumour and retrieval of specimens of either sufficient size or quantity (e.g. three to five biopsies for optimal tumour microenvironment assessment). In addition, staining for additional markers may also provide more comprehensive assessment; e.g. the addition of CD8^+^ could provide a biopsy‐based surrogate comparable to the Immunoscore 8, whereas staining for markers associated with tumour‐associated fibroblasts may improve sensitivity and specificity of the biopsy assessment of TSP.

Despite the above technical factors being considered, it was of interest that 14 tumours incorrectly classified as high CD3^+^ T‐cell density had technically satisfactory biopsy specimens. This may however reflect the semi‐quantitative nature employed for assessment of immune cell infiltrates within the surgically resected specimen, as 12 tumours had evidence of weak rather than absent CD3^+^ T‐cell infiltration. Therefore, it may be expected that a more quantitative and objective assessment of immune infiltrates within the resected specimen may have shown closer correlation between biopsy and surgically resected specimens.

In addition to allowing for preoperative assessment of the tumour microenvironment, biopsy‐based assessment was shown to have prognostic value independent of pathological staging. Using the combination of biopsy TSP and T‐lymphocyte density alone, it was possible to stratify five‐year survival from 92% (high CD3^+^ density) to 51% (low CD3^+^ density and high TSP), strikingly similar to the prognostic value of the GMS 6. Although it is not expected that biopsy‐based assessment of the tumour microenvironment will replace full section analysis following potentially curative surgery, it may however have a role in the treatment of patients not undergoing surgical resection. For example, it would be of considerable interest to investigate whether biopsy assessment of the tumour microenvironment may stratify survival of patients with advanced, inoperable colorectal cancer, and how this may subsequently impact upon therapeutic options. Furthermore, given recent interest in the neoadjuvant treatment of locally advanced colon cancer 27, as well as the potential application of total neoadjuvant therapy regimens, there will be a need for more detailed assessment of initial biopsy specimens in the event of complete clinical and pathological response.

The tumour microenvironment is a viable therapeutic target in patients with gastrointestinal cancers 28, 29, 30. The present work provides a justification for the use of biopsy‐derived assessments to quantify immune cell infiltrates and stromal infiltration on biopsies. Biopsy‐derived assessment may allow for personalisation of preoperative treatment strategies, particularly where the tumour microenvironment may be immune ‘cold’ or ‘altered’ 15. For example, in patients diagnosed with rectal cancer and found to have a low lymphocyte density on biopsy, one potential strategy could involve preoperative radiotherapy to promote tumour immunogenicity and increase tumour infiltration by activated immune cells 8, 9, 10, with serial biopsies performed to evaluate subsequent response. Taken together, it is clear that accurate biopsy‐derived assessments are key to the successful implantation of ‘window of opportunity’ trials in which novel neoadjuvant strategies tested prior to surgical resection are assessed for response based on *in vivo* biomarkers. We present a rationale for the use of the threshold of ≥25 cells/HPF to categorise CD3+ infiltrates as high or low grade in biopsies that has the potential to be applied in clinical studies.

The present study is limited by the lack of a validation cohort and use of a relatively historical cohort; whether the present results are reproducible in other cohorts and using more contemporary, targeted specimens of sufficient quality remains to be determined. However, the present methodologies employed may be readily tested and allow for validation in other cohorts of patients with colorectal cancer.

In conclusion, the results of the present study suggest that assessment and staging of the tumour microenvironment of patients undergoing resection of colorectal cancer is feasible using endoscopic biopsy specimens. This simple approach will allow for appropriate stratification of patients and treatment selections on the basis of the tumour microenvironment prior to potentially curative resection.

## Author contributions statement

JHP conceived the study design, performed data collection, analysis and interpretation, literature search, writing of the manuscript and generation of figures. HvW performed data analysis and interpretation. DCM, CSDR and JE conceived the study design and performed data interpretation and assisted with writing of the manuscript. CO assisted with data collection and analysis. PGH performed data interpretation and assisted with writing of the manuscript. All authors were involved in final approval of the submitted manuscript.

## Supporting information


**Figure S1.** Receiver operating characteristic curves displaying the relationships between biopsy intra‐epithelial CD3^+^ T‐cell count and full section assessment of CD3^+^ T‐cell density

**Table S1.** Sensitivity, specificity, positive and negative predictive values of biopsy‐based assessment of the tumour microenvironment of patients with primary operable colorectal cancer

**Table S2.** Relationships between full‐section assessment of tumour microenvironment, clinicopathological characteristics and cancer‐specific survival of patients with primary operable colorectal cancer

**Table S3.** Descriptive assessment of discrepancies between biopsy and full section assessment of T‐lymphocyte density

## References

[1] O'Connell JB , Maggard MA , Ko CY . Colon cancer survival rates with the new American joint committee on cancer sixth edition staging. J Natl Cancer Inst 2004; 96: 1420–1425. 15467030 10.1093/jnci/djh275

[2] Jass JR , Love SB , Northover JM . A new prognostic classification of rectal cancer. Lancet 1987; 1: 1303–1306. 2884421 10.1016/s0140-6736(87)90552-6

[3] Klintrup K , Makinen JM , Kauppila S , *et al* Inflammation and prognosis in colorectal cancer. Eur J Cancer 2005; 41: 2645–2654. 16239109 10.1016/j.ejca.2005.07.017

[4] Pagès F , Mlecnik B , Marliot F , *et al* International validation of the consensus Immunoscore for the classification of colon cancer: a prognostic and accuracy study. Lancet 2018; 391: 2128–2139. 29754777 10.1016/S0140-6736(18)30789-X

[5] Huijbers A , Tollenaar RA , v Pelt GW , *et al* The proportion of tumor‐stroma as a strong prognosticator for stage II and III colon cancer patients: validation in the VICTOR trial. Ann Oncol 2013; 24: 179–185. 22865778 10.1093/annonc/mds246

[6] Park JH , McMillan DC , Powell AG , *et al* Evaluation of a tumor microenvironment‐based prognostic score in primary operable colorectal cancer. Clin Cancer Res 2015; 21: 882–888. 25473000 10.1158/1078-0432.CCR-14-1686

[7] Hynes SO , Coleman HG , Kelly PJ , *et al* Back to the future: routine morphological assessment of the tumour microenvironment is prognostic in stage II/III colon cancer in a large population‐based study. Histopathology 2017; 71: 12–26. 28165633 10.1111/his.13181

[8] Anitei MG , Zeitoun G , Mlecnik B , *et al* Prognostic and predictive values of the immunoscore in patients with rectal cancer. Clin Cancer Res 2014; 20: 1891–1899. 24691640 10.1158/1078-0432.CCR-13-2830

[9] Shinto E , Hase K , Hashiguchi Y , *et al* CD8+ and FOXP3+ tumor‐infiltrating T cells before and after chemoradiotherapy for rectal cancer. Ann Surg Oncol 2014; 21: S414–S421. 24566864 10.1245/s10434-014-3584-y

[10] Teng F , Meng X , Kong L , *et al* Tumor‐infiltrating lymphocytes, forkhead box P3, programmed death ligand‐1, and cytotoxic T lymphocyte‐associated antigen‐4 expressions before and after neoadjuvant chemoradiation in rectal cancer. Transl Res 2015; 166: 721–732.e1. 26209749 10.1016/j.trsl.2015.06.019

[11] Yasuda K , Nirei T , Sunami E , *et al* Density of CD4(+) and CD8(+) T lymphocytes in biopsy samples can be a predictor of pathological response to chemoradiotherapy (CRT) for rectal cancer. Radiat Oncol 2011; 6: 49. 21575175 10.1186/1748-717X-6-49 PMC3120676

[12] Lim SH , Chua W , Cheng C , *et al* Effect of neoadjuvant chemoradiation on tumor‐infiltrating/associated lymphocytes in locally advanced rectal cancers. Anticancer Res 2014; 34: 6505–6513. 25368252

[13] Park JH , Richards CH , McMillan DC , *et al* The relationship between tumour stroma percentage, the tumour microenvironment and survival in patients with primary operable colorectal cancer. Ann Oncol 2014; 25: 644–651. 24458470 10.1093/annonc/mdt593 PMC4433525

[14] Kirilovsky A , Marliot F , El Sissy C , *et al* Rational bases for the use of the Immunoscore in routine clinical settings as a prognostic and predictive biomarker in cancer patients. Int Immunol 2016; 28: 373–382. 27121213 10.1093/intimm/dxw021 PMC4986234

[15] Galon J , Bruni D . Approaches to treat immune hot, altered and cold tumours with combination immunotherapies. Nat Rev Drug Discov 2019; 18: 197–218. 30610226 10.1038/s41573-018-0007-y

[16] Knudson KM , Hicks KC , Luo X , *et al* M7824, a novel bifunctional anti‐PD‐L1/TGFβ trap fusion protein, promotes anti‐tumor efficacy as monotherapy and in combination with vaccine. Oncoimmunology 2018; 7: e1426519. 29721396 10.1080/2162402X.2018.1426519 PMC5927523

[17] Mirjolet C , Charon‐Barra C , Ladoire S , *et al* Tumor lymphocyte immune response to preoperative radiotherapy in locally advanced rectal cancer: the LYMPHOREC study. Oncoimmunology 2018; 7: e1396402. 29399395 10.1080/2162402X.2017.1396402 PMC5790354

[18] Roxburgh CS , Shia J , Vakiani E , *et al* Potential immune priming of the tumor microenvironment with FOLFOX chemotherapy in locally advanced rectal cancer. Oncoimmunology 2018; 7: e1435227. 29872576 10.1080/2162402X.2018.1435227 PMC5980419

[19] Koelzer VH , Lugli A , Dawson H , *et al* CD8/CD45RO T‐cell infiltration in endoscopic biopsies of colorectal cancer predicts nodal metastasis and survival. J Transl Med 2014; 12: 81. 24679169 10.1186/1479-5876-12-81 PMC4022053

[20] Courrech Staal EF , Smit VT , van Velthuysen ML , *et al* Reproducibility and validation of tumour stroma ratio scoring on oesophageal adenocarcinoma biopsies. Eur J Cancer 2011; 47: 375–382. 21036599 10.1016/j.ejca.2010.09.043

[21] Richards CH , Roxburgh CS , Powell AG , *et al* The clinical utility of the local inflammatory response in colorectal cancer. Eur J Cancer 2014; 50: 309–319. 24103145 10.1016/j.ejca.2013.09.008

[22] Williams G , Quirke P , Shepherd NAN , *et al* Standards and Datasets for Reporting Cancers. Dataset for Colorectal Cancer (2nd edn). The Royal College of Pathologists: London, 2007.

[23] Jourdan F , Sebbagh N , Comperat E , *et al* Tissue microarray technology: validation in colorectal carcinoma and analysis of p53, hMLH1, and hMSH2 immunohistochemical expression. Virchows Arch 2003; 443: 115–121. 12802583 10.1007/s00428-003-0833-z

[24] Alderdice M , Richman SD , Gollins S , *et al* Prospective patient stratification into robust cancer‐cell intrinsic subtypes from colorectal cancer biopsies. J Pathol 2018; 245: 19–28. 29412457 10.1002/path.5051 PMC5947827

[25] van Krieken JH , Jung A , Kirchner T , *et al* KRAS mutation testing for predicting response to anti‐EGFR therapy for colorectal carcinoma: proposal for an European quality assurance program. Virchows Arch 2008; 453: 417–431. 18802721 10.1007/s00428-008-0665-y

[26] Saridaki Z , Saegart X , De Vriendt V , *et al* KRAS, NRAS, BRAF mutation comparison of endoscopic and surgically removed primary CRC paired samples: is endoscopy biopsy material adequate for molecular evaluation? Br J Cancer 2015; 113: 914–920. 26325103 10.1038/bjc.2015.307 PMC4578093

[27] Seymour MT , Morton D . FOxTROT: an international randomised controlled trial in 1052 patients (pts) evaluating neoadjuvant chemotherapy (NAC) for colon cancer. J Clin Oncol 2019; 37: abstr 3504.

[28] Sjoquist KM , Renfro LA , Simes RJ , *et al* Personalizing survival predictions in advanced colorectal cancer: the ARCAD nomogram project. J Natl Cancer Inst 2018; 110: 638–648. 29267900 10.1093/jnci/djx253 PMC6005015

[29] Whatcott CJ , Han H , Von Hoff DD . Orchestrating the tumor microenvironment to improve survival for patients with pancreatic cancer: normalization, not destruction. Cancer J 2015; 21: 299–306. 26222082 10.1097/PPO.0000000000000140 PMC4817719

[30] Moehler M , Delic M , Goepfert K , *et al* Immunotherapy in gastrointestinal cancer: recent results, current studies and future perspectives. Eur J Cancer 2016; 59: 160–170. 27039171 10.1016/j.ejca.2016.02.020

