# Supplementary figures and images for: Preoperative, biopsy‐based assessment of the tumour microenvironment in patients with primary operable colorectal cancer
Source: J Pathol Clin Res. 2019 Oct 14;6(1):30–9. doi: 10.1002/cjp2.143 (PMC6966701; doi:10.1002/cjp2.143)

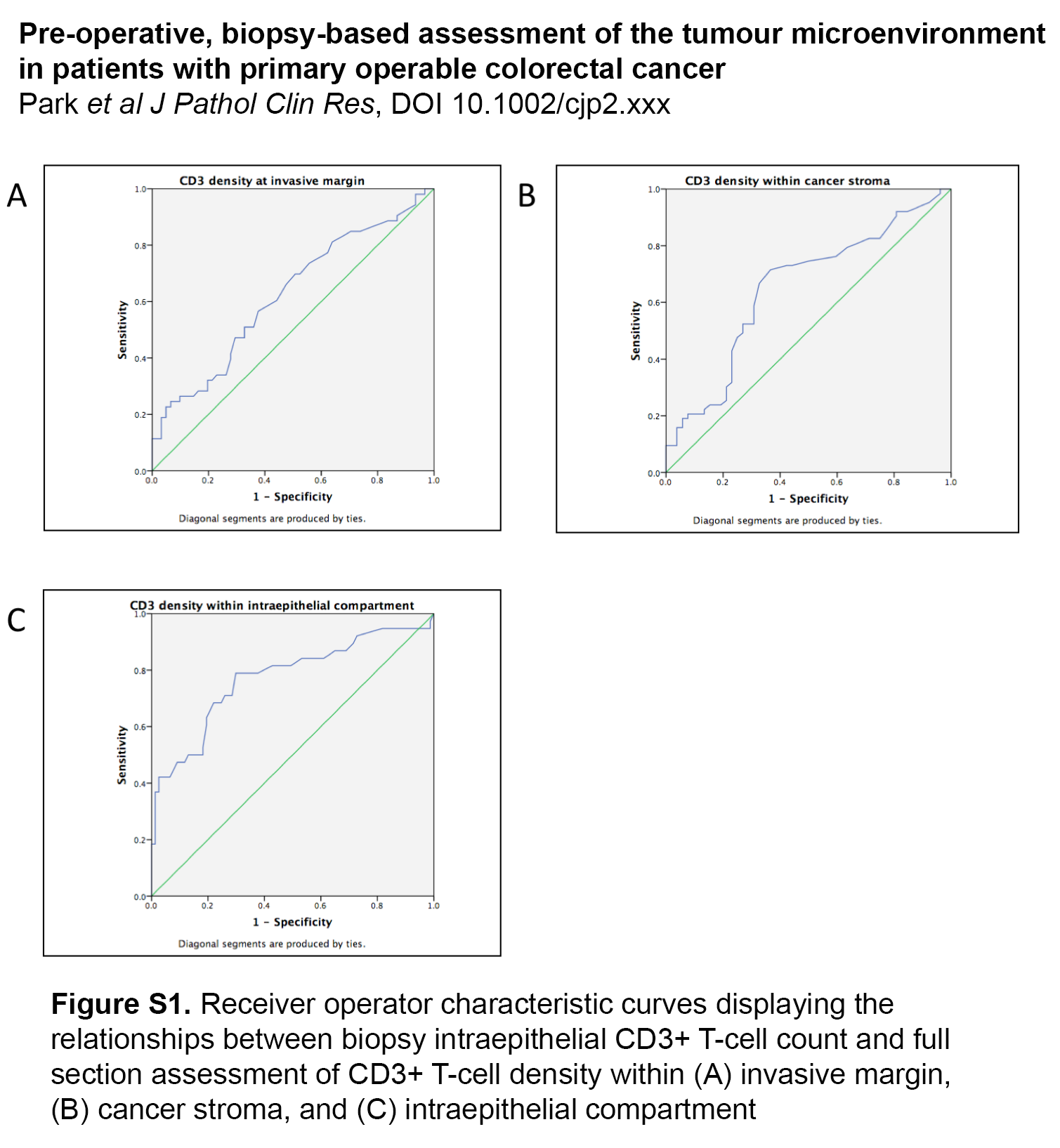

Supplement: Supplementary file 1 — Figure S1. Receiver operating characteristic curves displaying the relationships between biopsy intra‐epithelial CD3+ T‐cell count and full section assessment of CD3+ T‐cell density [file CJP2-6-30-s001.tiff]
