# Supplementary material for: Preoperative, biopsy‐based assessment of the tumour microenvironment in patients with primary operable colorectal cancer
Source: J Pathol Clin Res. 2019 Oct 14;6(1):30–9. doi: 10.1002/cjp2.143 (PMC6966701; doi:10.1002/cjp2.143)
Supplement: Supplementary file 2 — Table S1. Sensitivity, specificity, positive and negative predictive values of biopsy‐based assessment of the tumour microenvironment of patients with primary operable colorectal cancer [file CJP2-6-30-s002.docx]

**Pre-operative, biopsy-based assessment of the tumour microenvironment in patients with primary operable colorectal cancer**

Park JH *et al J Pathol Clin Res*, DOI 10.1002/cjp2.143

**Table S1.** Sensitivity, specificity, positive and negative predictive values of biopsy-based assessment of the tumour microenvironment of patients with primary operable colorectal cancer

|  | Biopsy CD3^+^ T-cell density | | | | | |
| --- | --- | --- | --- | --- | --- | --- |
| Full section CD3^+^ T-cell density | **Low** | **High** | **Sensitivity** | **Specificity** | **Positive predictive value** | **Negative predictive value** |
| CD3^+^ Margin  Low  High | 38  23 | 23  30 | 57% | 62% | 57% | 62% |
| CD3^+^ Stroma  Low  High | 36  26 | 16  37 | 59% | 69% | 70% | 58% |
| CD3^+^ Intraepithelial  Low  High | 54  8 | 23  30 | 79% | 70% | 57% | 87% |
|  | **Biopsy tumour stroma percentage** | | | | | |
| Full section TSP  Low  High | 64  9 | 26  16 | 64% | 71% | 38% | 90% |
