# Supplementary material for: Preoperative, biopsy‐based assessment of the tumour microenvironment in patients with primary operable colorectal cancer
Source: J Pathol Clin Res. 2019 Oct 14;6(1):30–9. doi: 10.1002/cjp2.143 (PMC6966701; doi:10.1002/cjp2.143)
Supplement: Supplementary file 3 — Table S2. Relationships between full‐section assessment of tumour microenvironment, clinicopathological characteristics and cancer‐specific survival of patients with primary operable colorectal cancer [file CJP2-6-30-s003.docx]

**Pre-operative, biopsy-based assessment of the tumour microenvironment in patients with primary operable colorectal cancer**

Park JH *et al J Pathol Clin Res*, DOI 10.1002/cjp2.143

**Table S2.** Relationships between full-section assessment of tumour microenvironment, clinicopathological characteristics and cancer-specific survival of patients with primary operable colorectal cancer

| **Full section assessment** | **Cancer-specific survival** | | | | | |
| --- | --- | --- | --- | --- | --- | --- |
| **Clinicopathological characteristics** |  | **Univariate analysis** | ***P*** |  | **Multivariate analysis** | ***P*** |
| **Age (<65/ 65-74/ >75)** |  | 1.17 (0.77-1.77) | 0.471 |  | - | - |
| **Sex (Female/ male)** |  | 1.41 (0.70-2.84) | 0.331 |  | - | - |
| **Adjuvant therapy (No/ yes)** |  | 1.21 (0.59-2.51) | 0.600 |  | - | - |
| **Tumour site (Colon/ rectum)** |  | 1.74 (0.86-3.50) | 0.123 |  | - | - |
| **TNM stage (I/ II/ III)** |  | 2.49 (1.25-4.93) | 0.009 |  | - | 0.179 |
| **Tumour differentiation (Mod-well/ poor)** |  | 1.50 (0.40-6.29) | 0.577 |  | - | - |
| **Venous invasion (No/ yes)** |  | 3.34 (1.66-6.70) | 0.001 |  | 3.97 (1.93-8.20) | <0.001 |
| **Margin involvement (No/ yes)** |  | 5.93 (2.05-17.11) | 0.001 |  | 6.92 (2.30-20.79) | 0.001 |
| **Peritoneal involvement (No/ yes)** |  | 1.69 (0.83-3.44) | 0.147 |  | - | - |
| **Tumour perforation (No/ yes)** |  | 3.10 (0.42-22.81) | 0.266 |  | - | - |
| **Full section intra-epithelial T-lymphocyte density (Low/ high)** |  | 0.22 (0.08-0.64) | 0.005 |  | 0.21 (0.07-0.61) | 0.004 |
| **Full section tumour stroma percentage (Low/ high)** |  | 2.41 (1.17-4.98) | 0.018 |  | 2.25 (1.07-4.73) | 0.033 |
