# Supplementary material for: Preoperative, biopsy‐based assessment of the tumour microenvironment in patients with primary operable colorectal cancer
Source: J Pathol Clin Res. 2019 Oct 14;6(1):30–9. doi: 10.1002/cjp2.143 (PMC6966701; doi:10.1002/cjp2.143)
Supplement: Supplementary file 4 — Table S3. Descriptive assessment of discrepancies between biopsy and full section assessment of T‐lymphocyte density [file CJP2-6-30-s004.docx]

**Pre-operative, biopsy-based assessment of the tumour microenvironment in patients with primary operable colorectal cancer**

Park JH *et al J Pathol Clin Res*, DOI 10.1002/cjp2.143

**Table S3.** Descriptive assessment of discrepancies between biopsy and full section assessment of T-lymphocyte density

| **Low Biopsy Density/ High Full Section Density (*n*=7)** | **High Biopsy Density/ Low Full Section Density (*n*=23)** |
| --- | --- |
| **Technical issues**   - 3 small biopsy/ fragmented specimen - 2 small invasive cancer foci within adenoma/ mucosa   **Non-technical**   - 2 no apparent technical issue | **Technical issues**   - 6 small biopsy/ fragmented specimen - 3 small invasive cancer foci within adenoma/ mucosa   **Non-technical**   - 14 no apparent technical issue   - 12 specimens had weak rather than absent full section CD3^+^ infiltration |
